# Reduced Genome of the Gut Symbiotic Bacterium “*Candidatus* Benitsuchiphilus tojoi” Provides Insight Into Its Possible Roles in Ecology and Adaptation of the Host Insect

**DOI:** 10.3389/fmicb.2020.00840

**Published:** 2020-05-06

**Authors:** Shakhinur Islam Mondal, Arzuba Akter, Ryuichi Koga, Takahiro Hosokawa, Mehmet Dayi, Kazunori Murase, Ryusei Tanaka, Shuji Shigenobu, Takema Fukatsu, Taisei Kikuchi

**Affiliations:** ^1^Division of Parasitology, Faculty of Medicine, University of Miyazaki, Miyazaki, Japan; ^2^Genetic Engineering and Biotechnology Department, Shahjalal University of Science and Technology, Sylhet, Bangladesh; ^3^Biochemistry and Molecular Biology Department, Shahjalal University of Science and Technology, Sylhet, Bangladesh; ^4^Bioproduction Research Institute, National Institute of Advanced Industrial Science and Technology, Tsukuba, Japan; ^5^Faculty of Science, Kyushu University, Fukuoka, Japan; ^6^Forestry Vocational School, Düzce University, Düzce, Turkey; ^7^NIBB Core Research Facilities, National Institute for Basic Biology, Okazaki, Japan; ^8^Department of Biological Sciences, Graduate School of Science, The University of Tokyo, Tokyo, Japan; ^9^Graduate School of Life and Environmental Sciences, University of Tsukuba, Tsukuba, Japan

**Keywords:** stinkbug symbiont, diapause, *Parastrachia japonensis*, Gammaproteobacteria, carotenoid synthesis, uric acid

## Abstract

Diverse animals, including insects, harbor microbial symbionts within their gut, body cavity, or cells. The subsocial parastrachiid stinkbug *Parastrachia japonensis* is well-known for its peculiar ecological and behavioral traits, including its prolonged non-feeding diapause period and maternal care of eggs/nymphs in an underground nest. *P. japonensis* harbors a specific bacterial symbiont within the gut cavity extracellularly, which is vertically inherited through maternal excretion of symbiont-containing white mucus. Thus far, biological roles of the symbiont in the host lifecycle has been little understood. Here we sequenced the genome of the uncultivable gut symbiont “*Candidatus* Benitsuchiphilus tojoi.” The symbiont has an 804 kb circular chromosome encoding 606 proteins and a 14.5 kb plasmid encoding 13 proteins. Phylogenetic analysis indicated that the bacterium is closely related to other obligate insect symbionts belonging to the Gammaproteobacteria, including *Buchnera* of aphids and *Blochmannia* of ants, and the most closely related to *Ishikawaella*, an extracellular gut symbiont of plataspid stinkbugs. These data suggested that the symbiont genome has evolved like highly reduced gamma-proteobacterial symbiont genomes reported from a variety of insects. The presence of genes involved in biosynthesis pathways for amino acids, vitamins, and cofactors in the genome implicated the symbiont as a nutritional mutualist, supplementing essential nutrients to the host. Interestingly, the symbiont’s plasmid encoded genes for thiamine and carotenoid synthesis pathways, suggesting the possibility of additional functions of the symbiont for protecting the host against oxidative stress and DNA damage. Finally, possible involvement of the symbiont in uric acid metabolism during diapause is discussed.

## Introduction

Many insects harbor symbiotic bacteria within their gut, body cavity, or cells (Buchner, 1965; Bourtzis and Miller, 2003; Engel and Moran, 2013). These symbiotic associations form a dynamic spectrum with regards to the necessity as well as the effects of the symbiont on the host (Baumann, 2005). Bacterial symbionts have various roles in host survival and reproduction, for instance providing essential nutrients, defense against natural enemies, participating in cuticle formation, increasing host digestive capacity, increasing host resistance against unfavorable environmental conditions, and detoxifying insecticides (Akman et al., 2002; Oliver et al., 2003; Russell and Moran, 2006; Kikuchi et al., 2012; Parker et al., 2013; Anbutsu et al., 2017; Salem et al., 2017). In obligate symbiotic associations, as a rule, symbionts are vertically transmitted to the next, where the symbiont transmission is integrated into the intricate development of the host insect (Braendle et al., 2003). Both the host and symbiont depend on each other for their development and reproduction (Wernegreen, 2002). Obligate symbiosis and strict maternal transmission tend to exhibit peculiar genomic characteristics such as drastic size reduction with AT-biased nucleotide content, accelerated sequence evolution and host–symbiont co-speciation (Shigenobu et al., 2000; Akman et al., 2002; Gil et al., 2003; Degnan et al., 2005; Nakabachi et al., 2006; Wu et al., 2006; McCutcheon and Moran, 2007, 2011; Nikoh et al., 2011; Kaiwa et al., 2014). In facultative symbiotic associations, by contrast, survival of the host insects is not dependent of their bacterial symbionts (Oliver et al., 2010), which exhibit the aforementioned genomic characteristics to a much lesser extent (Wu et al., 2004; Degnan et al., 2009; Klasson et al., 2009; Burke and Moran, 2011).

Parastrachiidae is a heteropteran stinkbug family containing one genus and two species, *Parastrachia japonensis* and *Parastrachia nagaensis* (Schaefer et al., 1991; Sweet and Schaefer, 2002). *P. japonensis* has interesting ecological and behavioral traits, including a prolonged (10 months to 2 years) non-feeding diapause at the adult stage, care of eggs in an underground nest, production of trophic eggs for consumption by newborn nymphs, and maternal collection and provision of food drupes from the *Schoepfia jasminodora* tree for the nymphs (Tachikawa and Schaefer, 1985; Tsukamoto and Tojo, 1992; Filippi et al., 2001; Hironaka et al., 2005). In a posterior region of the midgut of *P. japonensis*, sac-like structures, called crypts, develop to harbor an extracellular bacterial symbiont, “*Candidatus* Benitsuchiphilus tojoi” (hereafter referred to *Benitsuchiphilus* for simplicity) (Hosokawa et al., 2010). The symbiont is vertically transmitted via maternal excretion of symbiont-containing white mucus upon egg hatching, and likely to have an intimate host–symbiont association with biological importance over evolutionary time (Hosokawa et al., 2010, 2012). Also it has been suggested that the symbiont may be involved in uric acid recycling during diapause of *P. japonensis* (Kashima et al., 2006; Hosokawa et al., 2010).

In an attempt to gain insight into the evolutionary history and the biological roles of *Benitsuchiphilus* in the insect lifecycle, we sequenced the complete genome of the bacterium. Our comparative genome analysis of *Benitsuchiphilus* with other endocellular and extracellular symbiotic and free-living Gammaproteobacteria revealed the evolution of *Benitsuchiphilus* within the context of free-living and other symbiotic bacteria. Finally, the possibility that the symbiont is involved in uric acid metabolism during diapause is discussed.

## Results and Discussion

### Genomic Features of *Candidatus* Benitsuchiphilus tojoi

**FIGURE 1:**
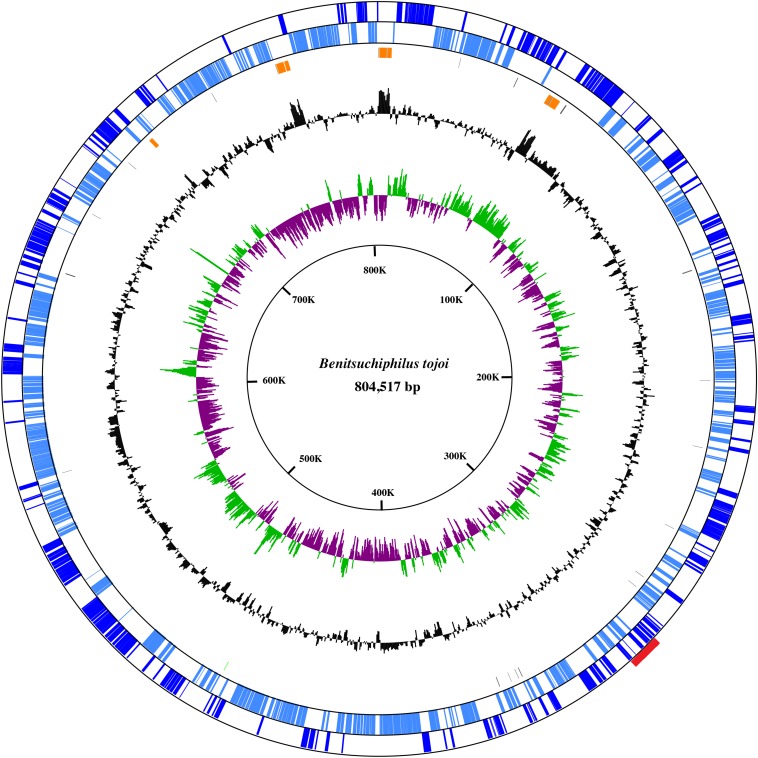
A circular view of the *Benitsuchiphilus* chromosome. The outermost circle and the second circle show the positions of the protein-coding genes in clockwise and counterclockwise directions, respectively. The third circle from the outside represents the position of rRNA, tRNA, and tmRNA. The innermost and second-innermost circles show the GC skew (green and purple) and the GC content (black), respectively. The GC-skew and GC-content values were calculated using a sliding-window (window size = 1,000 bp; step size = 500 bp). Red box in outermost circle indicates the phage region.

**FIGURE 2:**
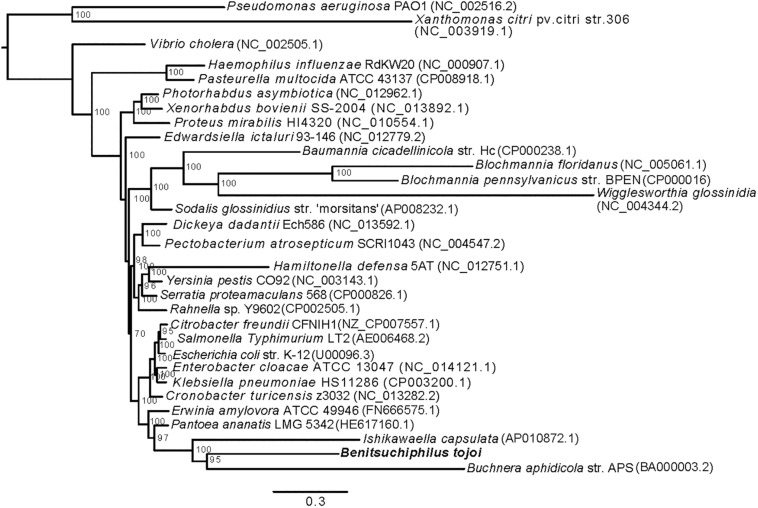
Phylogenetic placement of *Benitsuchiphilus* in Gammaproteobacteria. A maximum-likelihood tree was constructed using 49,559 amino acid positions from 166 one-to-one orthologous genes. Bar represents amino acid substitutions per site. Bootstrap values are shown at each node.

An adult female stinkbug (*Parastrachia japonensis*) was dissected and the symbiotic section of the posterior midgut was isolated. The midgut section is filled with *Benitsuchiphilus* cells (Hosokawa et al., 2010). Approximately 1 μg of total DNA was prepared and sequenced using PacBio and Illumina MiSeq sequencers to obtain 128 K and 9.4 M reads (with a mean length of 7.7 kb and 301 bp), respectively. Assembly of the PacBio reads resulted in one large (∼805 kb) and one small (15 kb) circular contigs with depth of coverage of 88x and 63x, respectively. About 4.1 M MiSeq reads (43.5% of the total reads) were aligned to the two contigs with a median depth of coverage of 864 and 1198, respectively. BLAST-based classification of the unaligned MiSeq reads (5.31 M) found that about 93% of them were unassigned (no similarity to any organisms in the database) and about 4 and 1% reads were similar to sequences of Rickettsiales bacteria and Insecta, respectively. These results indicated the isolated DNA contained the host insect DNA, whose sequences were scarcely available in the public database, and a small amount of *Wolbachia*-like bacterial DNA, in addition to the DNA of *Benitsuchiphilus.* Considering the high depths of coverage and the presence of a gene involved in replication in the small contig (Supplementary Table S1, please see below), the small circular contig was likely derived from a plasmid which is maintained at a small number of copies per chromosome in *Benitsuchiphilus* cell. The 804,517 bp *Benitsuchiphilus* chromosome, which was consistent with the previous genome size estimation of around 0.85 Mb (Hosokawa et al., 2010), encoded 606 protein-coding genes including 16 likely-truncated genes, 10 rRNA genes and 33 tRNA genes assigning all 20 standard amino acids (Figure 1 and Supplementary Table S1). The open reading frames (ORFs) of the 606 proteins had a median size of 962 bp, covering 72.5% of the whole genome. The plasmid was 14,499 bp in length and contained 13 ORFs (Supplementary Table S1). No tranposases, drug-resistance genes and IS elements were detected in the *Benitsuchiphilus* genome, but a prophage-like region of 11.5 kbp was found in the chromosome (Figure 1). The region contained 12 protein-coding sequences (CDSs) including 8 phage-related and four hypothetical genes (Supplementary Table S1). Those genes included a homolog of DNA polymerase III theta (HOT), recombination protein UvsY, and phage-related proteases but the region didn’t have phage-conserved genes including integrases, portal proteins, terminases, and tail tape measure proteins (Canchaya et al., 2003). To speculate the origin of the prophage-like regions, we have performed BLAST search of the eight phage-related genes and found that they are most similar to genes identified in *Pantoea* and *Erwinia* species (Supplementary Table S2), which were placed at the basal position of *Benitsuchiphilus*-containing cluster in the phylogenetic analysis (Figure 2, please see below), suggesting phage-related genes in *Benitsuchiphilus* have evolved from the same common ancestor of these bacterial species of the Erwiniaceae family.

The 805-kb *Benitsuchiphilus* genome was less than one-seventh the size of the genomes of free-living *Serratia proteamaculans* and *Escherichia coli* (Table 1). Almost all ORFs of *Benitsuchiphilus* (97.0%; 589/607) had orthologs in the genome of *S. proteamaculans*, indicating that the *Benitsuchiphilus* genome is a small subset of the free-living Gammaproteobacteria genome. The genome size of *Benitsuchiphilus* was in the range of other obligate gamma symbiotic bacteria including *Buchnera* of aphids (Shigenobu et al., 2000), *Blochmannia* of ants (Degnan et al., 2005), *Wigglesworthia* of tsetse flies (Akman et al., 2002), *Baumannia* of leafhoppers (Wu et al., 2006), and obligate extracellular symbionts of stinkbugs *Ishikawaella* (Nikoh et al., 2011) (Table 1). As with other obligate symbionts of insects, the *Benitsuchiphilus* genome had a reduced G + C% (27.93%) compared with free living bacteria, representing a base compositional bias in reduced bacterial genomes. Decreased GC content is also a hallmark characteristic of genome reduction that accompanies elevated fixation of mutations under relaxed selection in endosymbionts with stable host-restricted lifestyles (Moran et al., 2008; McCutcheon and Moran, 2011).

**TABLE 1:** General genomic features of the *Benitsuchiphilus*, extracellular insect symbionts, endocellular insect symbionts and free-living γ-proteobacteria.

**Strain**	***Benitsuchiphilus tojoi***	***Ishikawaella capsulata***	***Buchnera aphidicola***	***Baumannia cicadellinicola***	***Blochmannia pennsylvanicus***	***Wigglesworthia glossinidia***	***Sodalis glossinidius***	***Serratia proteamaculans***	***Escherichia coli K12***
GenBank accession number	PRJDB6854	AP010872.1	BA000003.2	CP008985.1	CP000016	NC_004344.2	AP008232.1	CP000826.1	U00096.3
Habitat	Extracellular Insect symbiont	Extracellular Insect symbiont	Intracellular Insect symbiont	Intracellular Insect symbiont	Intracellular Insect symbiont	Intracellular Insect symbiont	Insect symbiont	Free living	Free living
Host insect	Stinkbug (*Parastrachia japonensis*)	Stinkbug (*Megacopta punctatissima*)	Aphid (*Acyrthosiphon pisum*)	Sharpshooter (*Homalodisca coagulata*)	Ant (*Camponotus pennsylvanicus*)	Tsetse flies (*Glossina morsitans morsitans*)	Tsetse flies (*Glossina morsitans morsitans*)	Not applicable	Not applicable
Chromosome size (bp)	804,517	745,590	640,681	686,194	791,654	697,724	4,171,146	5,448,853	4,639,675
Plasmid	1	1	2	0	0	1	3	1	0
Prophage-related genes	12	ND	ND	ND	ND	ND	264	46	177
G + C content (%)	28	30	26	35	32	24	55	56	51
Coding content (%)	72.5	83	88	89	76.7	89	50.9	87	88
Total CDS	606 + 13	607 + 8	564 + 10	595	615	611 + 6	2432 + 54 + 23 + 7	5013 + 69	4,243
rRNAs	10	9	3	6	3	6	7	22	22
tRNAs	33	37	32	39	39	34	69	83	89
Other ncRNAs	0	3	4	2	2	2	5	11	74
Maximum protein size (AA)	1,408	1,416	1,408	1,409	1,417	1,406	1,485	3,603	2,358
Truncated/pseudo genes	16	36	13	9	4	14	972 + 23 + 5 + 3	83	99

*ND, not detected.*

**FIGURE 3:**
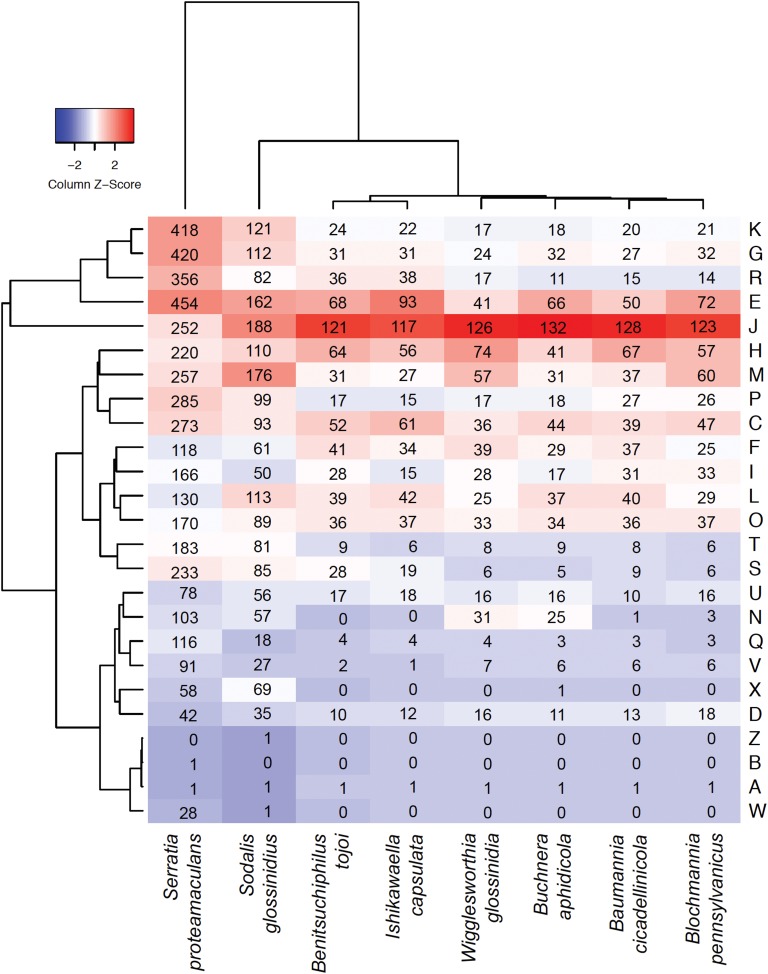
Heatmap comparison of the cluster of orthologous groups (COG) frequency profiles among *Benitsuchiphilus*, related symbiotic bacteria and the free-living *S. proteamaculans*. The dendrogram was generated using the *ggdendro* package. Numbers in cells indicate the number of genes in each COG category. Abbreviations for functional categories are as follows: J, translation; K, transcription; L, replication, recombination, and repair; D, cell cycle control; M, cell/wall membrane biogenesis; N, cell motility; O, post-translational modification, protein turnover, chaperones; P, inorganic ion transport and metabolism; T, signal transduction mechanism; U, intracellular trafficking and secretion; V, defense mechanism; C, energy production and conversion; E, amino acid transport and metabolism; F, nucleotide transport and metabolism; G, carbohydrate transport and metabolism; H, coenzyme transport and metabolism; I, lipid transport and metabolism; Q, secondary metabolite biosynthesis, transport, and catabolism; R, general function prediction only; and S, function unknown; X, mobilome: prophages, transposons; B, chromatin structure and dynamics; and W, extracellular structures.

**FIGURE 4:**
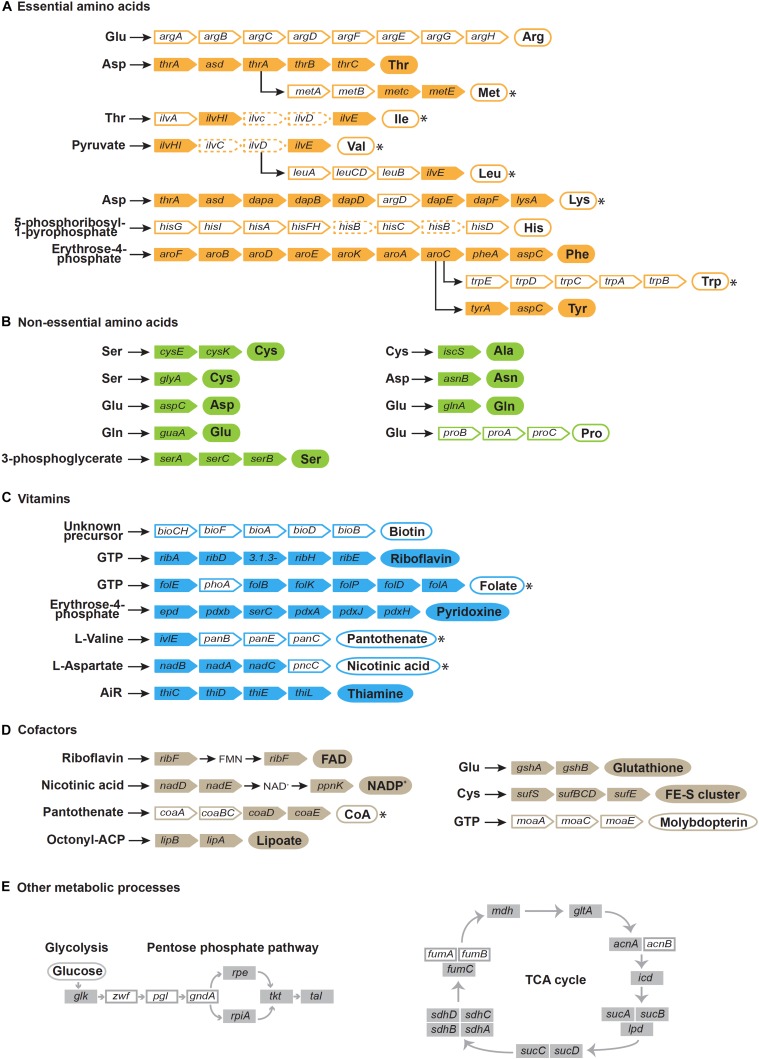
Reconstruction of pathways for the biosynthesis of essential amino acids **(A)**, non-essential amino acids **(B)**, vitamins **(C)**, cofactors **(D)**, and other metabolic processes **(E)**. Gene names are indicated in colored rectangles. White rectangles indicate missing genes, dashed rectangles indicate pseudogenes. Circles indicate products. Filled circles indicate synthetic pathways encoded in the *Benitsuchiphilus* genome that are complete and white circle indicate pathways that are incomplete, whereas circles with asterisks imply that the missing step/steps enzymes are probably complemented by other *Benitsuchiphilus* biosynthetic pathways, the host, or the host’s diet.

Although reduced, the *Benitsuchiphilus* genome still encoded genes involved in primary metabolic processes, aerobic respiration and the biosynthesis of peptidoglycan. The number of genes related to energy production and conversion in the *Benitsuchiphilus* genome was smaller than those in free-living Gammaproteobacteria but larger than those in endocellular symbiotic bacteria including *Blochmannia*, *Baumannia*, and *Wigglesworthia* (Figure 3). For example, the extracellular symbionts *Benitsuchiphilus* and *Ishikawaella* both retained genes for the complete TCA cycle, in contrast to the lack of those genes in endocellular symbionts (Figure 4) (Nikoh et al., 2011). This may be due to the evolutionary consequence of endocellular symbionts utilizing metabolic intermediates from the host cytoplasm, whereas extracellular symbionts have to retain their own metabolic genes.

Some intracellular insect symbionts with tiny genomes (e.g., *Hodgkinia* and *Tremblaya*) retain no genes involved in peptidoglycan biosynthesis and thus their cell wall is likely to be absent (McCutcheon and Moran, 2011). The presence of peptidoglycan synthesis genes in the *Benitsuchiphilus* genome (Supplementary Figure S1) suggested that the bacterium could produce cell walls which would be important for the persistence of symbiont cells in the extrachorion matrix. However, *Benitsuchiphilus* retained fewer cell wall synthesis genes involved in outer membrane construction than other endocellular symbiotic bacteria (Figure 3); *Benitsuchiphilus* does not retain biosynthetic pathways of lipopolysaccharides, which is common in symbionts with attenuated virulence or to avoid host immunologic responses (Kim et al., 2015). This might also be related to the fact that the host insect *P. japonensis* produces white mucus to compose the mucosa/bacteria mixture to provide nutritional support to newborn nymphs (Hosokawa et al., 2012). That mucus may have a role in symbiont protection under ex-host conditions.

*Benitsuchiphilus* has no genes for locomotion/motility like *Ishikawaella*, reflecting an exclusively symbiotic lifestyle in which these functions are plausibly not necessary in the host environment. However, the endocellular symbionts *Buchnera* and *Wigglesworthia* have retained many cell motility genes (mainly for flagellar basal bodies) (Shigenobu et al., 2000; Akman et al., 2002) (Figure 3). These genes are expressed and the proteins they encode are suspected to be used as material transporters to maintain the symbiotic system (Shigenobu et al., 2000; Akman et al., 2002; Maezawa et al., 2006; Rio et al., 2012). Like the extracellular symbiont *Ishikawaella, Benitsuchiphilus* is harbored extracellularly and may not require such transporters or flagellar motors.

Another feature typical of symbiotic bacteria, likely linked to the host-restricted lifestyle, is the maximal protein length reduction compared to free-living bacteria (Charles et al., 1999). In the *Benitsuchiphilus* genome, the largest protein-coding gene was 1,408 amino acids and encoded a beta subunit of RNA polymerase (RpoC) (Table 1 and Supplementary Figure S2). This was approximately one-third of the longest protein (3,602 amino acids) encoded by the genome of a free-living relative *S. proteamaculans*, which is involved in filamentous hemagglutinin adhesion. The proteins that are involved in virulence, toxin production, secondary metabolic processes, extracellular sensing, or of unknown function are generally the longest protein sequences in the proteome of free-living bacteria; they are largely missing in the insect symbiont genomes as these genes could no longer be required in a stable environment and are likely to contribute to their reduced genome size (Kenyon and Sabree, 2014).

### Phylogenetic and Gene Family Analyses

Inferring phylogenetic relationships of symbiotic bacteria among other bacteria is generally difficult because of higher rates of substitution in symbiont linkagesand A + T biased nucleotide compositions (Kikuchi et al., 2009; Philippe and Roure, 2011). In this study, we followed the tree construction procedure to reduce those long branch effects as described in Husnik et al. (2011) (see section “Materials and Methods). We used 166 one-to-one orthologous genes of 32 Gammaproteobacterial species (Figure 2). The resulting maximum-likelihood tree showed polyphyly of symbiotic Gammaproteobacteria as was seen previously (Husnik et al., 2011; Philippe and Roure, 2011). *Benitsuchiphilus* formed a cluster with symbiotic bacteria *Ishikawaella* and *Buchnera*, and plant pathogenic bacteria *Pantoea ananatis* and *Erwinia amylovora* (*Erwinia* cluster). *Ishikawaella* is an extracellular symbiont of stinkbugs of the family Plataspidae with a lifestyle similar to *Benitsuchiphilus* in that they colonize to the cavity of the crypt-bearing posterior midgut. Although previous studies suggest independent multiple origins of stinkbug symbionts in general (Hosokawa et al., 2016), our phylogenetic analysis suggested a rather close relationship of the two symbionts. *Buchnera*, an endocellular symbiont of aphids, was clustered with *Benitsuchiphilus*, however, its position remains uncertain as the recent study showed that *Buchnera* belongs to the Sodalis group which includes other endocellular symbiotic bacteria including *Blochmannia*, *Baumannia*, and *Wigglesworthia* (McCutcheon et al., 2019).

**FIGURE 5:**
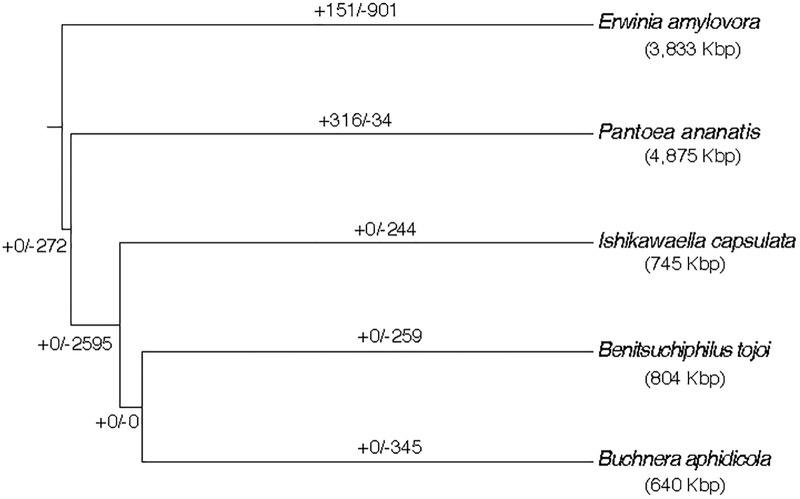
Estimation of ancestral gene gains and losses in the evolutionary course of *Benitsuchiphilus*, *Ishikawaella*, *Buchnera*, *P. ananatis*, and *E. amylovora*. The phylogenetic tree is a part of Figure 2. Numbers above the branches indicate gene gain and loss events. The genome size of each strain is indicated in parentheses.

Gene family evolution analysis was performed with the 32 Gammaproteobacterial species and the result of the *Erwinia* cluster is shown in Figure 5. A large gene loss was detected in the lineage where the three symbiotic bacteria (<1 Mb genomes) diversified from the common ancestor to *P. ananatis* (∼4.9 Mb genome). Large gene losses were also detected in the tail branches of the three symbionts and very low gene family losses/gains were shared by any two of the three symbionts. This suggested that the symbiont gene family losses occurred continuously and independently in each lineage after the diversification from the common ancestor of those and *P. ananatis.* The number of lost genes estimated for the *Benitsuchiphilus* branch was 259, which contained genes with a wide variety of biological functions, enriched with such GO terms as secondary metabolite biosynthetic process, biosynthesis of amino acids, carbohydrate metabolic process and bacterial-type flagellum assembly (Supplementary Table S3).

**FIGURE 6:**
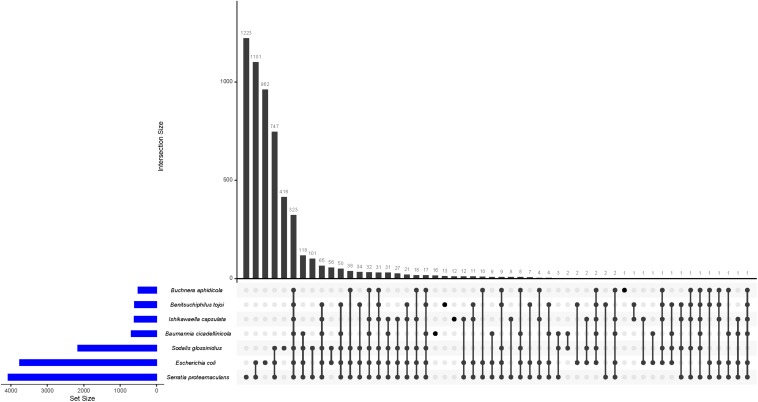
UpSet plot of the distribution of ortholog families in *Benitsuchiphilus*, *Ishikawaella*, *Buchnera*, *Baumannia*, *Sodalis*, *S. proteamaculans*, and *E. coli.* The plots show the number of shared ortholog families between genomes by the bar chart.

Next, we compared presence/absence of gene families in symbiotic bacteria (three from the Erwinia group; *Benitsuchiphilus*, *Ishikawaella, Buchnera* and two from the Sodalis group; *Baumannia* and *Sodalis*) with free-living bacteria (*S. proteamaculans* and *E. coli* selected as the representatives). While 323 gene families (orthogroups) were shared by all the seven species, no orthogroups were unique to the symbiotic bacteria (i.e., shared by the five symbiotic bacteria but not present in the free-living bacteria) (Figure 6). Only a small number of orthogroups were species-specific in each symbiotic bacterium. For instance, *Benitsuchiphilus, Ishikawaella, Buchnera*, and *Baumannia* had 13, 12, 1, and 16 species-specific orthogroups, respectively. The orthogroups unique to the *Benitsuchiphilus* genome included five genes that were presumably in a process of ongoing pseudogenization due to unnecessity in the lifestyle. Those genes encode truncated multimodular transpeptidase-transglycosylase (*mrcB*), lysyl-lysine 2,3-aminomutase (*epmB*), ketol-acid reductoisomerase (*ilvC*), dihydroxy-acid dehydratase (*ilvD*). The rest of the *Benitsuchiphilus*-specific genes were of unknown function with amino acid length of 69–114 aa. *Ishikawaella* contained 12 species-specific orthogroups; *ubiD* (3-octaprenyl-4-hydroxybenzoate decarboxylase) and 11 genes of unknown function. All the *Buchnera* and *Baummania* specific genes encode hypothetical proteins. These results support the hypothesis that the reduced symbiotic genomes tend to retain core genes dedicated to maintaining the symbiotic relationship and the central informational machinery (Moran and Bennett, 2014). It also suggested that a small number of species-specific genes are present in reduced symbiotic genome, which may play important roles in the specific biology/life styles of each symbiont.

To identify gene order conservation, we aligned the whole genomes of six symbiotic bacteria. The gene order was not highly conserved between any of the six genomes (Supplementary Figure S3), indicating that genome reductions in those symbiotic bacteria may have occurred along with genome rearrangements. This is consistent with the fact that the *Benitsuchiphilus* genome, like *Ishikawaella* and *Baumannia*, harbors several rRNA clusters and does not present a clear GC skew pattern (Figure 1), which indicates recent genome rearrangements and the machinery is possibly still active in the *Benitsuchiphilus* genome.

### Features of Putative Nutritional Mutualism and Supplementation

*Benitsuchiphilus* encoded a limited set of amino acid synthesis pathways. Complete sets of genes for the threonine, phenylalanine and tyrosine biosynthetic pathways were present in *Benitsuchiphilus*, whereas there were no genes found for the arginine, proline, and histidine biosynthetic pathways. One or more genes were missing in the methionine, lysine, tryptophan, valine, leucine, and isoleucine biosynthetic pathways (Figure 4). Missing enzymes or corresponding metabolites may be supplied by other *Benitsuchiphilus* biosynthetic pathways, the host, or the host’s diet. The transport system sometimes helps to compensate for the lack of amino acid synthesis pathways by importing amino acids from the environment (Tomii and Kanehisa, 1998; Wu et al., 2006). Though we found four genes associated with amino acid transport in *Benitsuchiphilus* (*fliY*, *yecC*, *yecS*, and *brnQ*), no specific transporters for the missing amino acids were found in the genome.

Although free-living bacteria synthesize many amino acids, intracellular pathogens/symbionts lose the ability over time to synthesize amino acids (Payne and Loomis, 2006). This is due to the high transcriptional and translational cost of amino acid biosynthetic pathways, and less selective pressure to synthesize products that can be provided by the host cell. However, despite the extreme genome reduction, many obligate mutualists (e.g., *Ishikawaella*, *Buchnera*, *Sulcia*, etc.) show the tendency to retain these pathways to supply amino acids to their hosts (Shigenobu et al., 2000; McCutcheon and Moran, 2007; Nikoh et al., 2011). Meanwhile, some obligate mutualists *Baumannia* and *Wigglesworthia* have a very limited capacity to synthesize amino acids for their hosts (Akman et al., 2002; Wu et al., 2006). It was reported that *Baumannia* and *Sulcia* are co-primary symbionts which co-inhabit the bacteriomes of host insects. This co-residence allows for the possibility of metabolic complementarity (Wu et al., 2006). In contrast, *Wigglesworthia* likely has a different reason; *Wigglesworthia*’s host food (vertebrate blood) is highly rich in amino acids (Akman et al., 2002; Rio et al., 2012).

The *Benitsuchiphilus* genome encoded 44 proteins putatively involved in the synthesis of a diverse set of vitamins, cofactors, prosthetic groups, and related compounds (Figure 4). Metabolic pathway reconstruction showed that complete pathways for *de novo* synthesis of riboflavin (vitamin B2), pyridoxine (vitamin B6), thiamine (vitamin B1) and the cofactors FAD, NADP^+^, lipoate, glutathione, FE-S cluster and molybdopterin were present in the *Benitsuchiphilus* genome. *Benitsuchiphilus* also seemed to have the ability to produce endogenously important precursors such as guanosine triphosphate, erythrose-4-phosphate, aspartate and 5-aminoimidazole ribonucleotide. The pathways for biosynthesis of folate (vitamin B9) and nicotinic acid (vitamin B3) were not complete due to a single missing gene (*phoA* and *pncC*, respectively). It is possible that the function encoded by *phoA* is performed instead by a non-specific phosphatase (Snyder and Rio, 2015) or that *phoA* may not be required at all (Bermingham and Derrick, 2002). The functions of *pncC* could be performed by other deamidases encoded by the *Benitsuchiphilus* genome. However, the three pathways for the synthesis of biotin (vitamin B7), panthothenate (vitamin B5) and molybdopterin were absent in the *Benitsuchiphilus* genome (Figure 4). Interestingly, we identified three genes for the heme biosynthetic pathway (*hemF*, *hemG*, and *hemH*) but the genes for the upstream processes were not identified, indicating that *Benitsuchiphilus* imports intermediate substrates to complete the heme biosynthetic pathway (Wu et al., 2006). Although the completeness of vitamin and cofactor synthesis pathways varied greatly, the presence of those pathways supports the hypothesis that *Benitsuchiphilus* provides these compounds to the host to compensate for their low abundance in their diet, in a similar way to other symbiotic bacteria, including *Baumannia* and *Wigglesworthia*, where more genes are retained for vitamin and cofactor synthesis (Akman et al., 2002; Wu et al., 2006).

The host of *Benitsuchiphilus, P. japonensis*, feeds solely on the drupe endosperm of a specific tree, *Schoepfia jasminodora*, for 2 weeks a year as its food source (Nomakuchi et al., 1998; Filippi et al., 2000). We currently do not explicitly know the nutritional content of the drupe, although it is likely that it contains a limited set of amino acids and vitamins/cofactors based on reports on the nutritional contents of other plant endosperms (Pirgozliev et al., 2003). Further study to analyze the detailed nutritional contents of the food will clarify the nutritional contribution of *Benitsuchiphilus* to the symbiotic system.

### *Benitsuchiphilus* Plasmid Encoded Genes for Carotenoid and Thiamine Biosynthesis

The *Benitsuchiphilus* plasmid (pBTOJ01) encoded 13 proteins including *crtE*, *crtY*, *crtI*, and *crtB* involved in the carotenoid biosynthesis pathway, *thiS, thiF, and thiG* in the thiamine biosynthesis pathway, a small heat shock protein *ibpB*, an ubiquinone/terpenoid-quinone biosynthesis protein *ubiXD*, a putative replication protein *repA* and two hypothetical proteins (Supplementary Table S1).

Carotenoids are a widespread class of pigments that absorb light and prevent oxidation (Sandmann, 2002; Fraser and Bramley, 2004), and thus play a variety of important functional roles. They are synthesized by diverse lineages of eubacteria, archaea, protists, fungi, and plants; however, animals generally cannot produce the precursors of carotenoids. In some insects, endosymbiotic bacteria provide a source of carotenoids (Sloan and Moran, 2012). The major genes involved in carotenoid biosynthesis are geranylgeranyl diphosphate synthase (*crtE*), phytoene synthase (*crtB*), phytoene desaturase (*crtI*), and lycopene cyclase (*crtY*). The *Benitsuchiphilus* plasmid had all the genes necessary for the *de novo* synthesis of carotenoids, likely providing a source of carotenoids to the host insect, as previously hypothesized in other insects (Sloan and Moran, 2012). Further analyses are required to assign precise functional roles of *Benitsuchiphilus*-derived carotenoids but it is speculated that they may function for vision and display coloration like in other animals (Britton et al., 2004) and/or protect the host against oxidative stresses by supplying it with reducing power (Santos-Garcia et al., 2012; Sloan and Moran, 2012). In addition, carotenoids have been found to protect against DNA damage and maintain genomic stability (Collins, 2001; Sloan and Moran, 2012). Facultative endosymbionts of aphids have been shown to affect host pigmentation through biochemical pathways unrelated to carotenoids (Tsuchida et al., 2010). Therefore, currently not recognized, the possible role of carotenoids in host pigmentation cannot be excluded.

**FIGURE 7:**
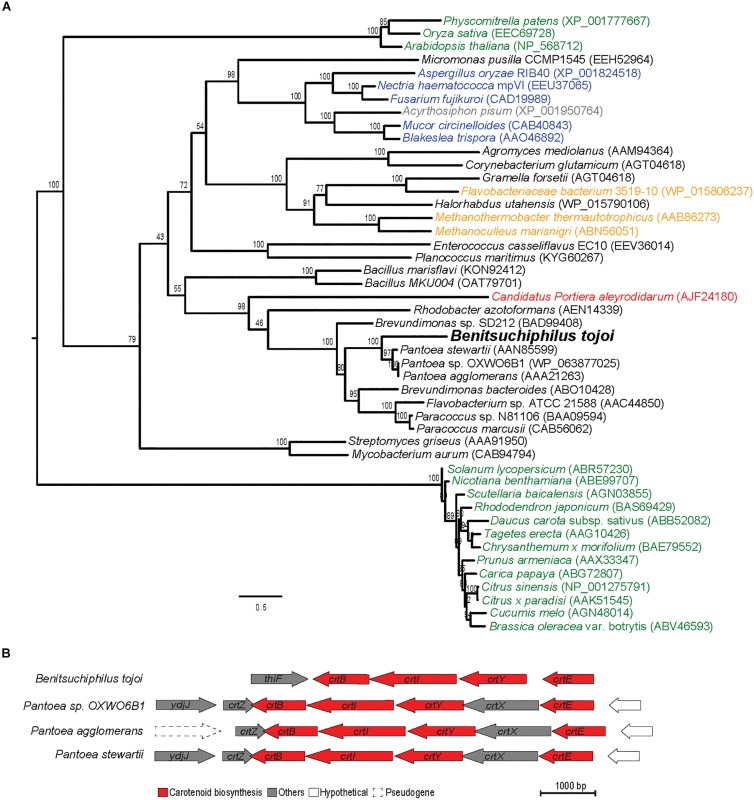
Maximum-likelihood protein phylogenies of carotenoid desaturase enzyme and the comparison of carotenoid biosynthesis genes of phylogenetically close species. **(A)** The phylogeny is shown with full taxon labeling. Colors indicate major groups, with bacteria in black, archaea in orange, plants in green, fungi in blue, aphids in gray and symbiont bacteria in red. Numbers on the branches represent the support from 1,000 bootstrap replicates. The scale bar indicates substitutions per site. **(B)** Structural organization of carotenoid biosynthesis genes and functionally unrelated flanking genes in *Benitsuchiphilus* and phylogenetically close *Pantoea* species. Carotenoid biosynthesis genes are red colored.

To identify the possible origin of genes for carotenoid biosynthesis in *Benitsuchiphilus*, phylogenetic analyses of phytoene desaturase (*crtI*) and phytoene synthase (*crtB*) genes with those found in bacteria, archaea, plants and fungi were conducted. Although lateral gene transfers of those genes from fungi were proposed in aphids and spider mites (Moran and Jarvik, 2010; Altincicek et al., 2012), our phylogenetic analyses showed that the carotenoid biosynthesis genes in *Benitsuchiphilus* were the most similar to those in the plasmids of Enterobacteriaceae bacteria (*Pantoea* spp.) (Figure 7A and Supplementary Figure S4). The *Pantoea* plasmids contain all genes necessary for *de novo* synthesis of carotenoids as a cluster, and the gene order of the four genes were conserved between the *Benitsuchiphilus* and *Pantoea* plasmids although the *Pantoea* plasmids possess additional genes in the cluster, including *crtZ* (3-β-ionone hydroxylase) and *crtX* (zeaxanthin glucosyl transferase), involved in further modification of beta-carotene (Figure 7B).

In addition, the pBTOJ01 plasmid contained three genes (*thiF, thiS*, and *thiG*) for thiamine biosynthesis (Supplementary Table S1). In *E. coli*, six structural proteins ThiF, ThiS, ThiG, ThiH, IscS, and ThiI play an important role in the production of the intermediate thiazole moiety from tyrosine, cysteine, and 1-deoxy-D-xylulose-5-phosphate in thiamine biosynthesis (Begley et al., 1999). We found the other two proteins (IscS and ThiI) are encoded by the chromosomal genes, but thiazole synthase, ThiH, which is used for the formation of the thiazole moiety from tyrosine, was missing *Benitsuchiphilus*, indicating cysteine and 1-deoxy-D-xylulose-5-phosphate as the possible source of thiamine biosynthesis. The other important genes (*thiC, D, E*, and *L*) for thiamine biosynthesis were also encoded by the chromosome. ThiC and ThiD are involved in pyrimidine biosynthesis from aminoimidazole ribotide, ThiE is required for the linking of the thiazole and the pyrimidine, and ThiL catalyzes the formation of the active form of thiamine. Generally, insects are not able to produce thiamine *de novo* (Sweetman and Palmer, 1928; Craig and Hoskins, 1940). Therefore, the plasmid genes, in cooperation with the chromosomal genes, may participate in *de novo* thiamine biosynthesis in the host for adaptation that required due to the nutrient deficiency in diet. Phylogenetic analyses of the genes on the plasmid reveled that they are closely related to genes found on plasmids of *Pantoea* and *Erwina* species (Supplementary Figures S5D,G,H) whereas the chromosomal genes were closely related to genes on the chromosomes of *Pantoea* and *Erwinia* (Supplementary Figures S5A–C,E,F and Table S4). This results suggest that the cooperative relationship of chromosomal and plasmid genes for thiamine synthesis has been retained from the ancestor of *Benitsuchiphilus, Pantoea* and *Erwinia* species and further supports the close phylogenetic relationship of those species.

### Possible Role in Uric Acid Metabolism

The host insect *P. japonensis* survives a long time (10 months to 2 years) without feeding. It is suggested that uric acid recycling plays an important role in this long term survival by utilizing nutritional stores (Kashima et al., 2006). The predominant nitrogenous waste product detected in *P. japonensis* is uric acid (Kashima et al., 2006). Uric acid is metabolized to urea through the uricolytic pathway with the aid of three enzymes (urate oxidase [or uricase], allantoinase, and allantoicase). Urea is then degraded by urease, generating ammonia and CO_2_. The released ammonia is assimilated by glutamine synthetase to glutamine and then to other amino acids (Kashima et al., 2006). Although it was previously shown that *Erwinia*-like bacteria associated with *P. japonensis* likely possess all of the uricolytic pathway enzymes (Kashima et al., 2006), the *Benitsuchiphilus* genome encoded for urate oxidase and glutamine synthetase but not the other three enzymes (allantoinase, allantoicase, and urease). It was observed that many insects depend on endosymbionts to recycle their nitrogenous waste products (Sasaki and Ishikawa, 1995; Hansen and Moran, 2011; Macdonald et al., 2012). Therefore, uric acid recycling in the insect *P. japonensis* may be completed by a combination of enzymes from *Benitsuchiphilus*, the insect host and/or other bacterial associates.

### Positively Selected Genes in the *Benitsuchiphilus* Genome

Positive selection is a mechanism by which new advantageous genetic variants sweep through a population and drive adaptive evolution. To investigate the roles of positive selection in the evolution of *Benitsuchiphilus*, we performed dN/dS analyses with a branch-site model using single-copy orthologous genes of 23 bacterial species. Forty-eight genes were detected as positively selected in the *Benitsuchiphilus* lineage (Supplementary Table S5). Intriguingly, the positive selection list was dominated with genes involved in amino acid metabolism, including tRNA ligases (10 genes), amino acid synthesis enzymes such as CTP synthase, adenylosuccinate synthetase, and tRNA(Ile)-lysidine synthase. These results might be related to the fact that *Benitsuchiphilus* lacks several amino acid synthesis pathways which is probably compensated by the host insect or its foods. Though the possibility of pseudo-positive detection of selected genes due to pseudogenization cannot be completely excluded, there might be host-mediated selections of relevant genes in symbionts as a result of genetic basis of host specialization. Other positively selected genes included DNA nucleases (RecBCD enzyme subunit genes), cell wall biogenesis (UDP-*N*-acetylmuramoylalanine–D-glutamate ligases).

## Conclusion

Genome analysis of the extracellular stinkbug symbiont *Benitsuchiphilus* revealed typical signatures of strong host association and vertical inheritance, including significant genome reduction, AT richness, retention of mutualism-supportive genes and an elevated evolutionary rate. Genome-based phylogeny and gene family analyses suggested that the symbiont genome is closely related to other gammaproteobacterial insect symbionts, but the evolution to a symbiont was likely independent. Although reduced, the *Benitsuchiphilus* genome still encoded genes involved in primary metabolic processes, aerobic respiration and the production of peptidoglycan, reflecting their adaptation to the extracellular environment. Metabolic pathway reconstruction indicated that *Benitsuchiphilus* is a nutritional mutualist, supplementing essential nutrients to the host. A plasmid of *Benitsuchiphilus* encoded thiamine and carotenoid biosynthesis pathway genes, suggesting additional symbiont roles to protect the host against oxidative stress and DNA damage. Albeit incomplete, the presence of genes involved in the uricolytic pathway in *Benitsuchiphilus* genome suggested some roles in uric acid recycling in the host, *P. japonensis*, during long-term diapause.

## Materials and Methods

### Insect Samples Collection and Genomic DNA Preparation

A female adult stinkbug (*Parastrachia japonensis*) was collected in Hinokuma Mountain Prefectural Park, Saga, Japan, and dissected in sterile phosphate-buffered saline (PBS) (0.8% NaCl, 0.02% KCl, 0.115% Na_2_HPO_4_, 0.02% KH_2_PO_4_ [pH 7.5]), using fine forceps and microscissors, by which the alimentary tract was isolated. Genomic DNA was extracted from the organ using QIAamp DNA Mini Kit (Qiagen).

### Genome Assembly and Annotation

The *Benitsuchiphilus* DNA was sequenced on the Illumina MiSeq and PacBio platform. Pair-end Illumina libraries were generated using the Nextera XT DNA sample prep kit (Illumina) following the manufacture’s instruction and sequenced to generate 301 bp pair-end reads using MiSeq v3-600 kit (Illumina). Low quality trimming and adapter removal for the Illumina reads were performed using Trimmomatic (Bolger et al., 2014) with the options (SLIDINGWINDOW:4:15 LEADING:3 TRAILING:3 MINLEN:180). To produce PacBio long reads, *Benitsuchiphilus* DNA was mixed with the same amount of a reference DNA (*Serratia marcescens* Db11 genomic DNA) and sequenced with SMRT cell 8Pac V3 and DNA Polymerase Binding Kit P6 (PacBio) using PacBio RSII system. The reads were filtered using the RS_Subreads protocol (minimum subread length = 1 kb, minimum polymerase read quality = 0.8), resulting in a total of 970 Mb useable data (total number of subreads = 128 K reads, mean subread length = 8.0 kb, subread N50 = 11.4 kb, subreads standard deviation = 5.4 kb).

The PacBio reads were assembled using Falcon (v.0.3.0^1^) and the assembly was improved using the Quiver module in the SMRT Analysis pipeline (version 2.3.0) (Chin et al., 2013) using the default settings. The sequence data possibly contained the host and/or other bacterial DNA in addition to *Benitsuchiphilus* genomic DNA, but the meta-assembly resulted in three contigs only including one from the reference DNA. When each end of contigs was overlapped, contigs were connected to form a circular DNA. The circularity was verified using Illumina paired-end reads mapped to the joint regions. Further base correction was then performed with the 5 Gb Illumina pair-end reads using ICORN2 (Otto et al., 2010) with five times iterations to produce the final genome assembly.

The *Benitsuchiphilus* genome was annotated using the Prokaryotic Genome Annotation System (PROKKA) (Seemann, 2014) and the Rapid Annotations using Subsystems Technology (RAST) server (Aziz et al., 2008) using the default settings. Finally, the annotation was manually curated using *in silico* Molecular Cloning software (In-silico biology). Easyfig (Sullivan et al., 2011) was implemented for gene order comparison of six symbiotic bacteria. For mobile genetic elements profiles, the prophages and insertion sequences (ISs)/transposons were predicted using PHASTER (Arndt et al., 2016) and IS Finder (Siguier et al., 2006) with default parameter, respectively.

### Gene Family Analyses and Molecular Phylogenetic Tree Construction

Orthologous protein groups from the proteomes of related Gammaproteobacteria with complete and draft genomes were determined using OrthoFinder v1.0.7 (Emms and Kelly, 2015). A total of 166 one-to-one orthologs from 32 species were used for species phylogenetic tree construction. Proteins of each ortholog group were aligned by using MAFFT version v7.221 with E-INS-I option (Katoh and Standley, 2013) and poorly aligning regions were trimmed using GBlocks v0.91b with the default options (Castresana, 2000). The resulted alignments were concatenated and used to generate a maximum-likelihood phylogenetic tree using IQ-TREE 2 (Minh et al., 2020) multicore version 2.0-rc1 v2.0 with the PMSF (posterior mean site frequency) model that was suggested to effectively ameliorate long branch attraction artifacts (Wang et al., 2018) with combination of LG + C10 + F + G with 100 bootstrap after generating a guide tree by LG + F + G model. Maximum-likelihood trees for carotenoid and thiamine biosynthesis genes were constructed using IQ-TREE with the same settings as outlined above. The best-supported maximum-likelihood tree was visualized using FigTree v1.3.1^2^. Gene family evolution analysis was performed using the OrthoFinder results with CAFÉ (Han et al., 2013) with the ultrametric tree generated by r8s (Sanderson, 2003) based on the species maximum-likelihood tree.

### Positive Selection Scanning in the *Benitsuchiphilus* Lineage

The tree used for positive selection analyses was a subset of the main phylogenetic tree to include 23 species from *Edwardsiella ictaluri* to *Benitsuchiphilus* (Figure 2). Amino acid sequences of 217 one-to-one orthologous genes from those 23 species were aligned using MAFFT with default options (Katoh and Standley, 2013) and were back-translated into nucleotide sequences using PAL2NAL with default options (Suyama et al., 2006). The resulting codon alignments were manually checked to filter out low quality alignments and only good alignments were used in the CODEML program of the ete3 toolkit (Huerta-Cepas et al., 2016) to calculate the rate of non-synonymous and synonymous substitution (dN/dS ratio) using branch-site model with bsA (for the alternative model) and bsA1 (for the null model) to test positive selection events against a neutral selection on a specific branch to background branch (ete3 options; – models bsA bsA1 – resume – clear_tree – mark Benitsuchiphilus). The alternative and null hypotheses were tested with three independent runs for each hypothesis and the best likelihood value of each run was kept to calculate the log-likelihood value (Dos Reis and Yang, 2011). The alternative model was contrasted to the null model using a likelihood-ratio test (LRT) in which log-likelihood ratios were compared to a chi-square distribution with 1 degree of freedom. The *P*-values of the successive comparisons were corrected for multiple testing by applying a FDR correction using qvalue R package (Dabney et al., 2010).

### Functional Categorization and Pathway Reconstruction

The predicted protein-coding genes were categorized on the basis of Cluster of Orthologous groups (COGs) functional classification (Tatusov et al., 2003). The COG profile displays were made using the Heatmapper (Babicki et al., 2016). Metabolic pathways were predicted and reconstructed based on the metabolic information from the Kyoto Encyclopedia of Genes and Genomes (KEGG) using blastKOALA (Kanehisa et al., 2016). Metabolic pathways were re-examined and verified according to the pathway descriptions in the EcoCyc (Keseler et al., 2013) and MetaCyc (Caspi et al., 2016) databases.

## Data Availability Statement

The genome reads, assemblies and annotation were deposited in the DNA Data Bank of Japan (DDBJ) Sequence Read Archive under the BioProject ID PRJDB6854.

## Author Contributions

TF and TK conceived the study. SM, AA, MD, KM, TK, and RK analyzed genome data. SM, AA, TF, and TK wrote the manuscript with input from others. RT, TH, and SS prepared materials and performed sequencing.

## Conflict of Interest

The authors declare that the research was conducted in the absence of any commercial or financial relationships that could be construed as a potential conflict of interest.

## Footnotes

1 https://github.com/PacificBiosciences/FALCON-integrate

2 http://tree.bio.ed.ac.uk/software/figtree/
