# Supplementary material for: Reduced Genome of the Gut Symbiotic Bacterium “Candidatus Benitsuchiphilus tojoi” Provides Insight Into Its Possible Roles in Ecology and Adaptation of the Host Insect
Source: Front Microbiol. 2020 May 6;11:840. doi: 10.3389/fmicb.2020.00840 (PMC7218078; doi:10.3389/fmicb.2020.00840)
Supplement: Supplementary file 1 [file Data_Sheet_1.pdf]

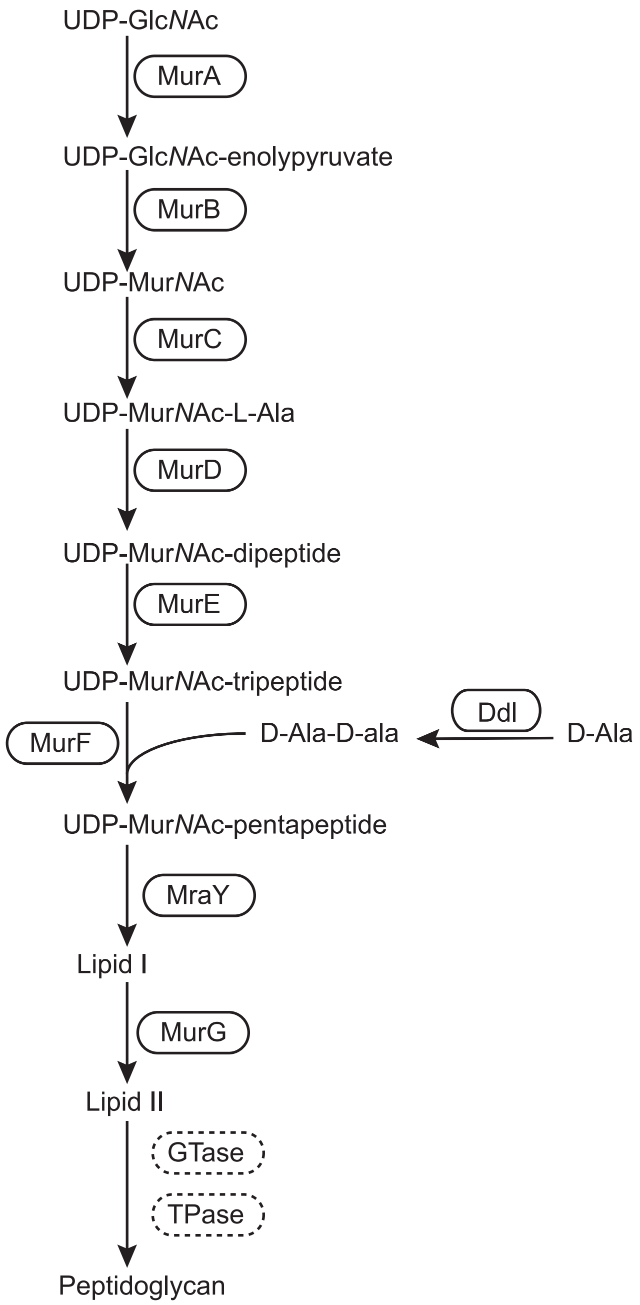

Supplementary Figure S1. Reconstruction of pathways for the biosynthesis of peptidoglycans in *Benitsuchiphilus*. *Benitsuchiphilus* genome retains all genes necessary to synthesise peptidoglycans.

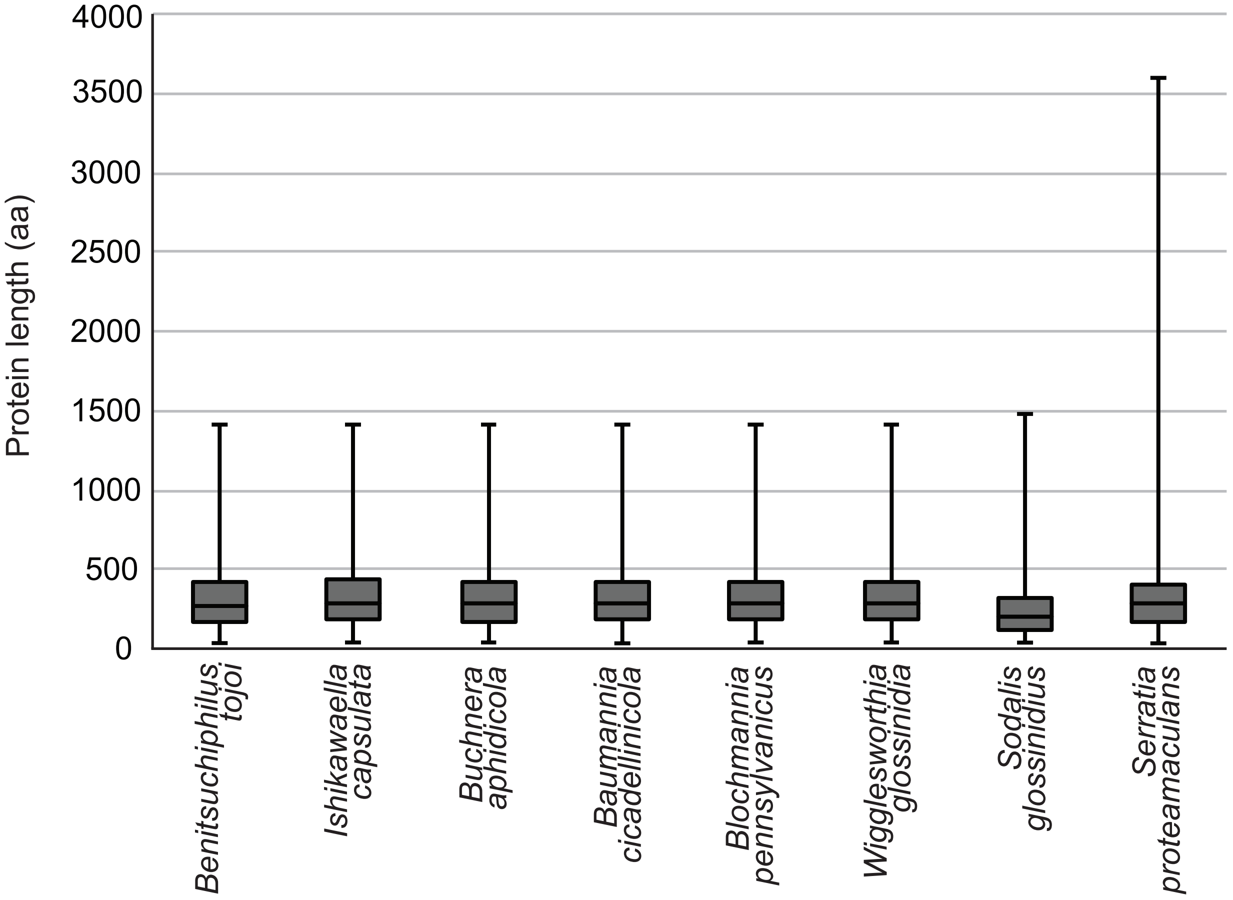

Supplementary figure S2. Protein length of *Benitsuchiphilus*, related symbiont bacteria and the free-living *S. proteamaculans.* Box plot illustrating maximum protein length reduction in symbiotic bacteria compare to free living relatives. The bottom and top of the box are the first and last quartiles and the line within the box is the 50th percentile (the median) of the points in the protein length.

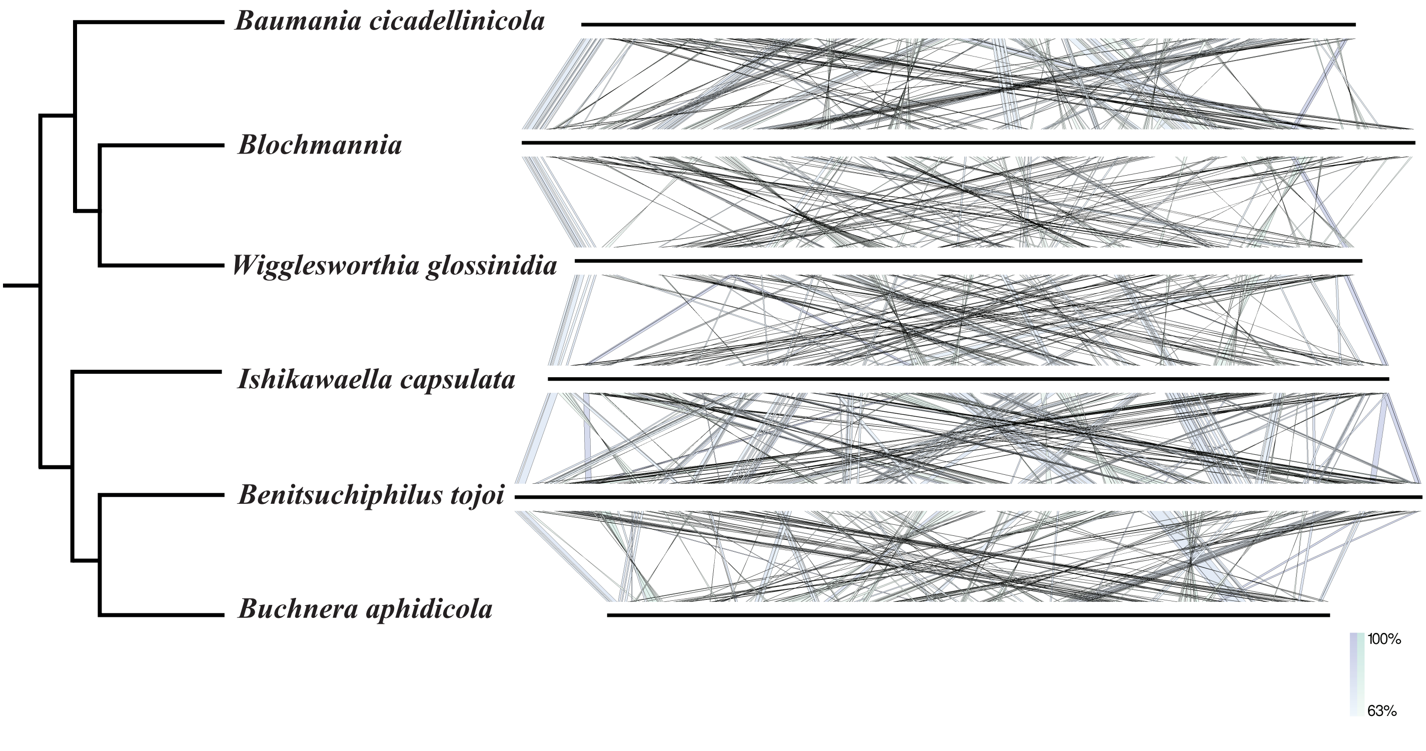

Supplementary Figure S3. Gene order comparison of six symbiotic bacteria.

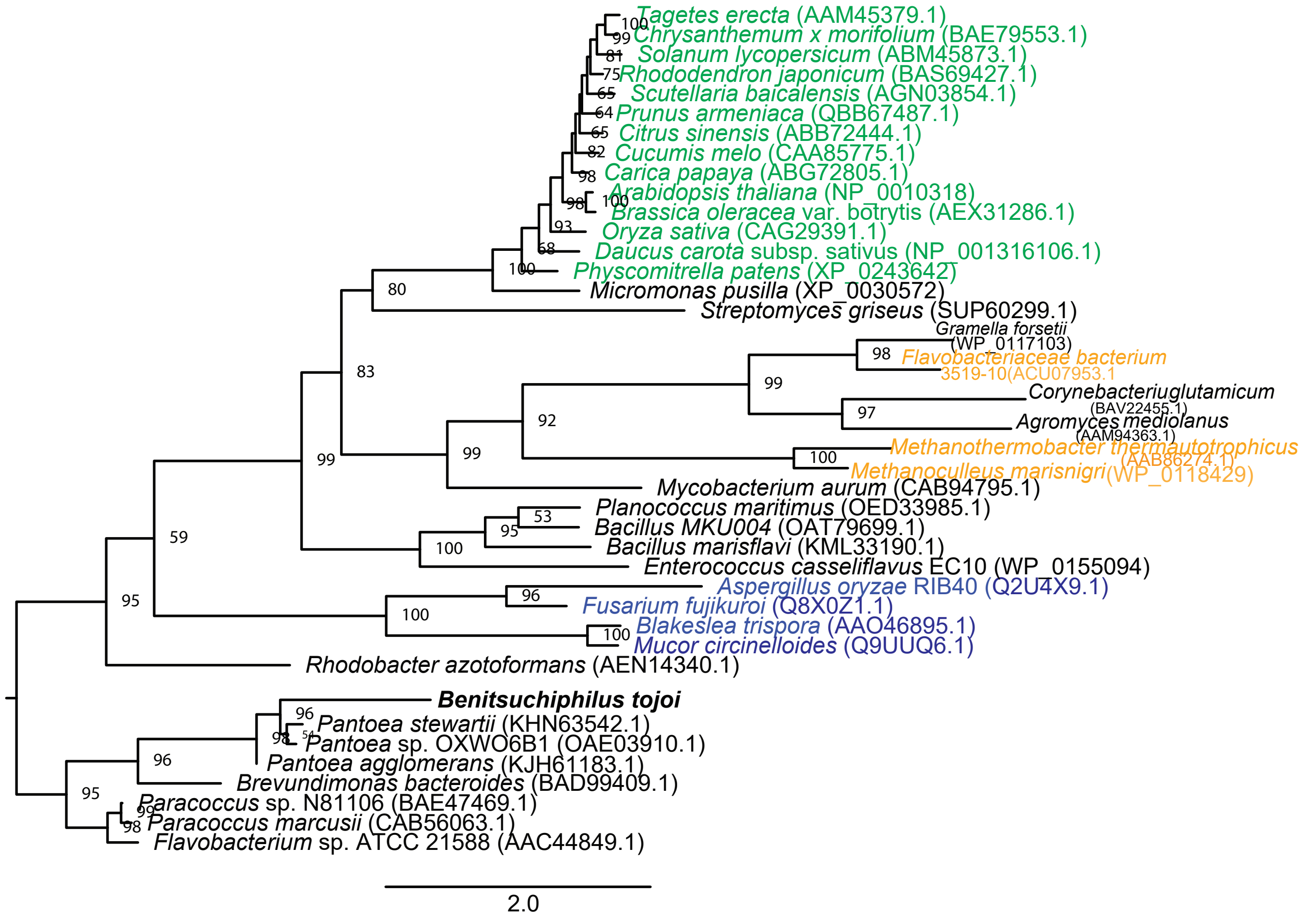

Supplementary Figure S4. Maximum-likelihood protein phylogenies of carotenoid synthase enzyme. The phylogeny is shown with full taxon labeling. Colors indicate major groups, with bacteria in black, archaea in orange, plants in green and fungi in blue. Numbers on the branches represent the support from 1,000 bootstrap replicates. The scale bar indicates substitutions per site.

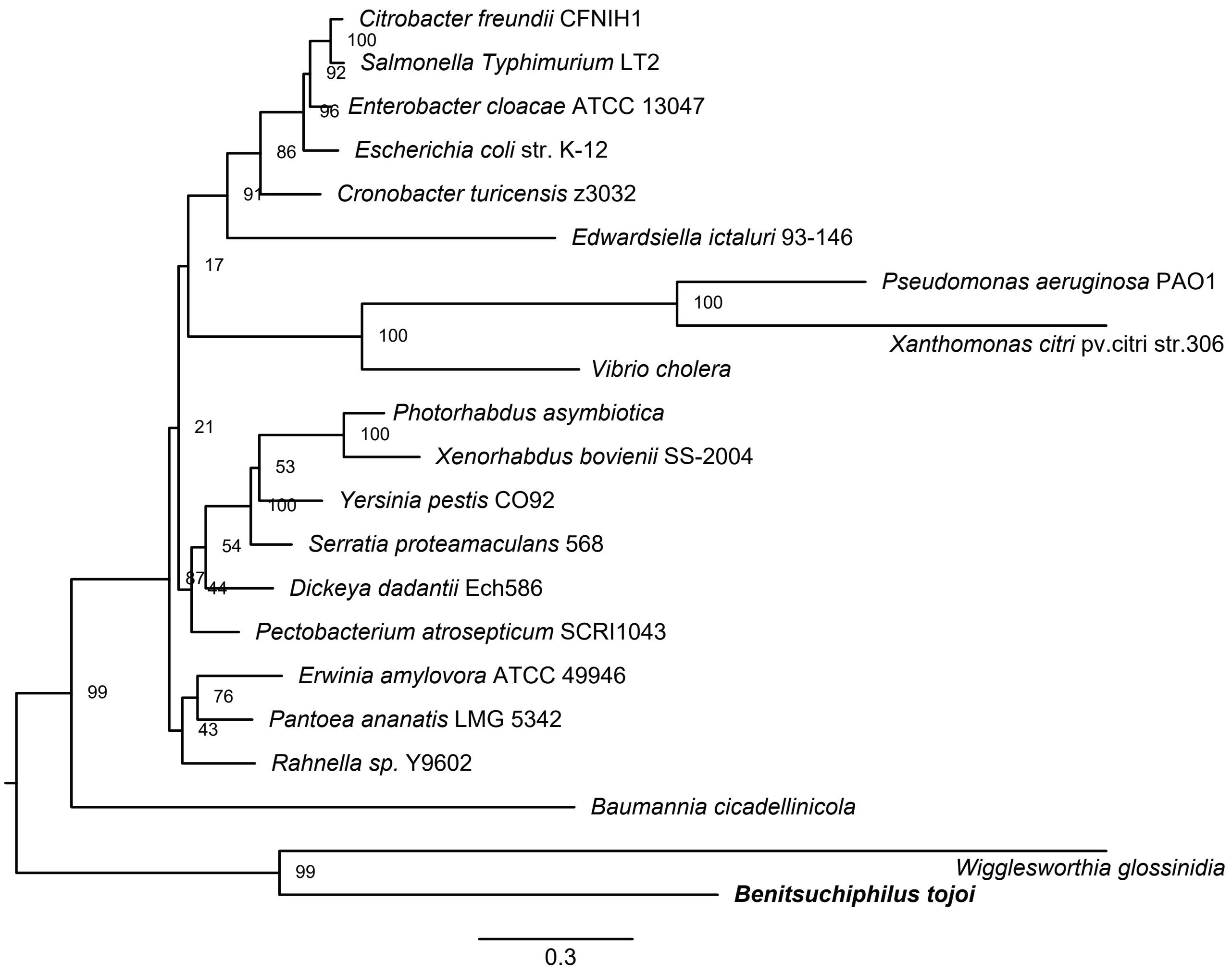

Supplementary Figure S5A. Maximum-likelihood protein phylogenies of ThiC. Numbers on the branches represent the support from 1,000 bootstrap replicates. The scale bar indicates substitutions per site.

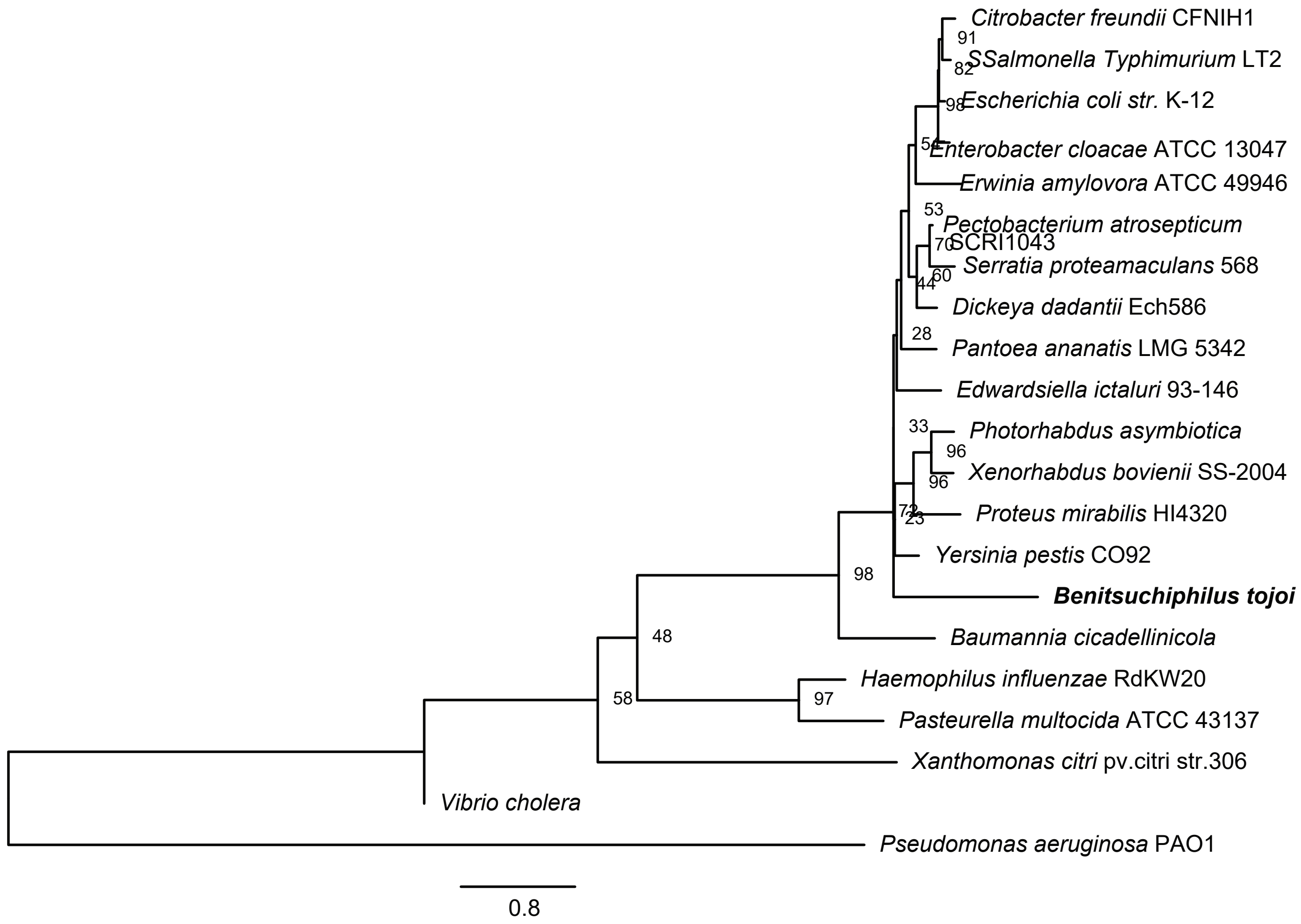

Supplementary Figure S5B. Maximum-likelihood protein phylogenies of ThiD. Numbers on the branches represent the support from 1,000 bootstrap replicates. The scale bar indicates substitutions per site.

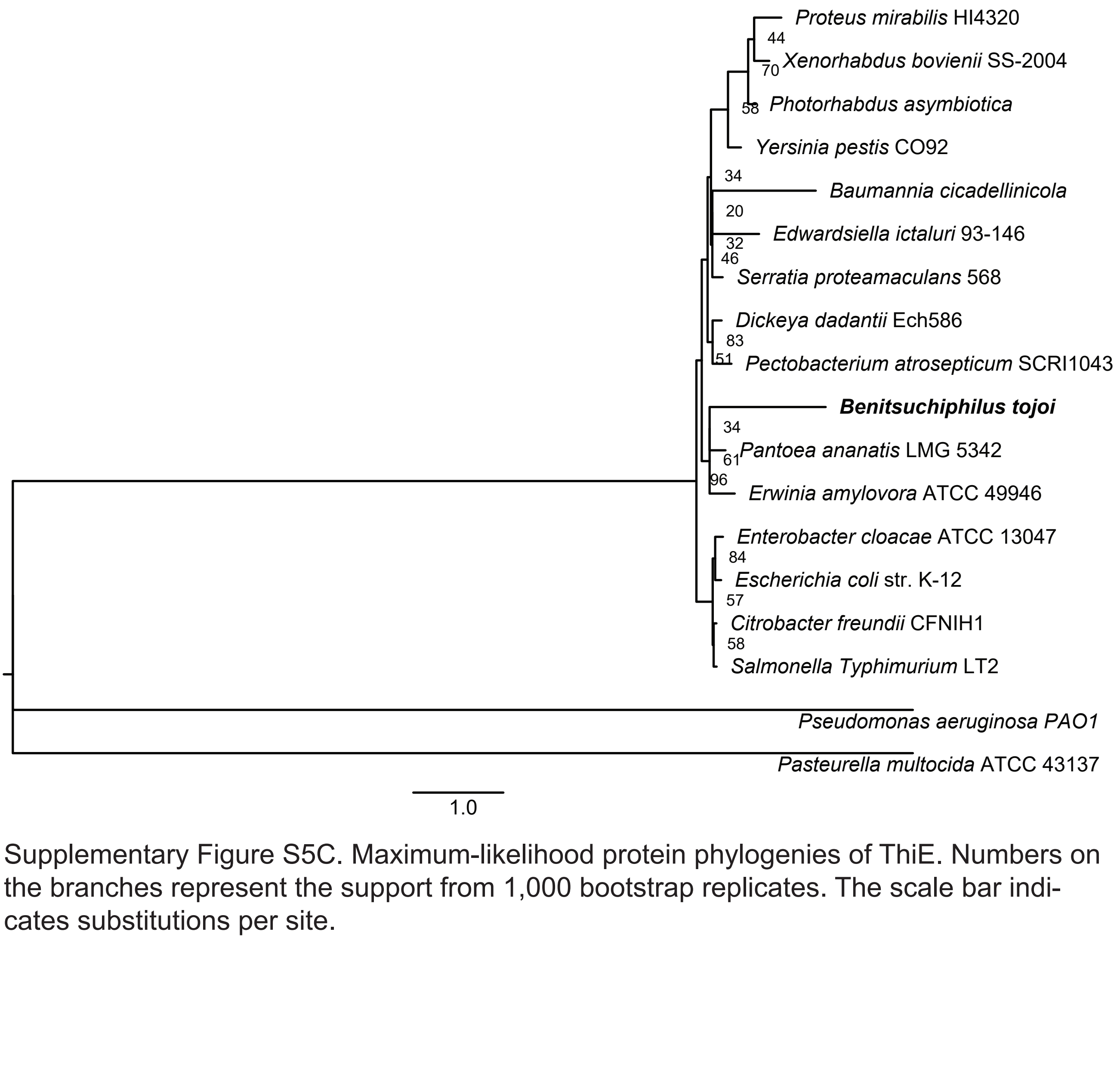

Supplementary Figure S5C. Maximum-likelihood protein phylogenies of ThiE. Numbers on the branches represent the support from 1,000 bootstrap replicates. The scale bar indicates substitutions per site.

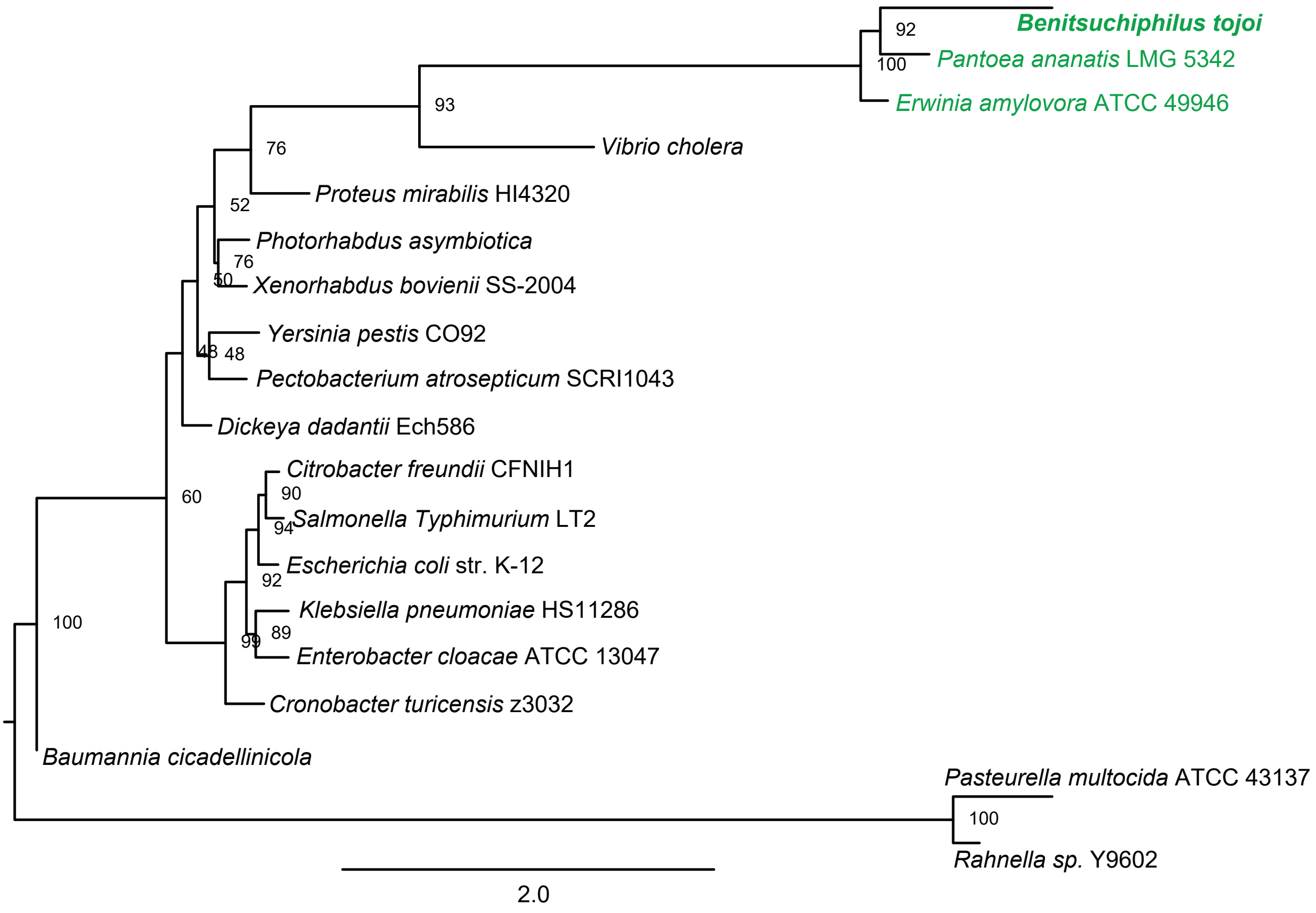

Supplementary Figure S5D. Maximum-likelihood protein phylogenies of ThiF. Green color indicate plasmid origin. Numbers on the branches represent the support from 1,000 bootstrap replicates. The scale bar indicates substitutions per site.

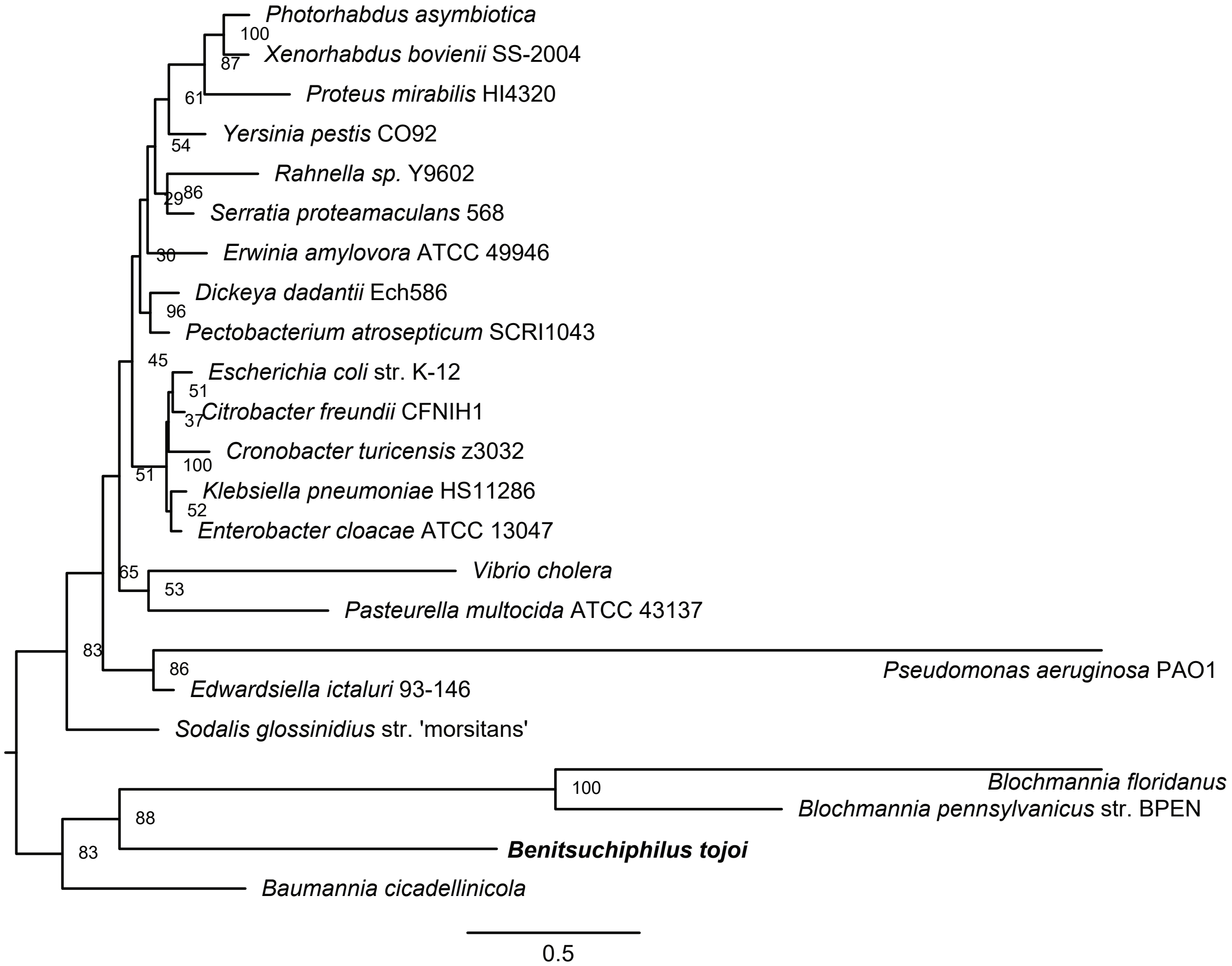

Supplementary Figure S5E. Maximum-likelihood protein phylogenies of ThiI. Numbers on the branches represent the support from 1,000 bootstrap replicates. The scale bar indicates substitutions per site.

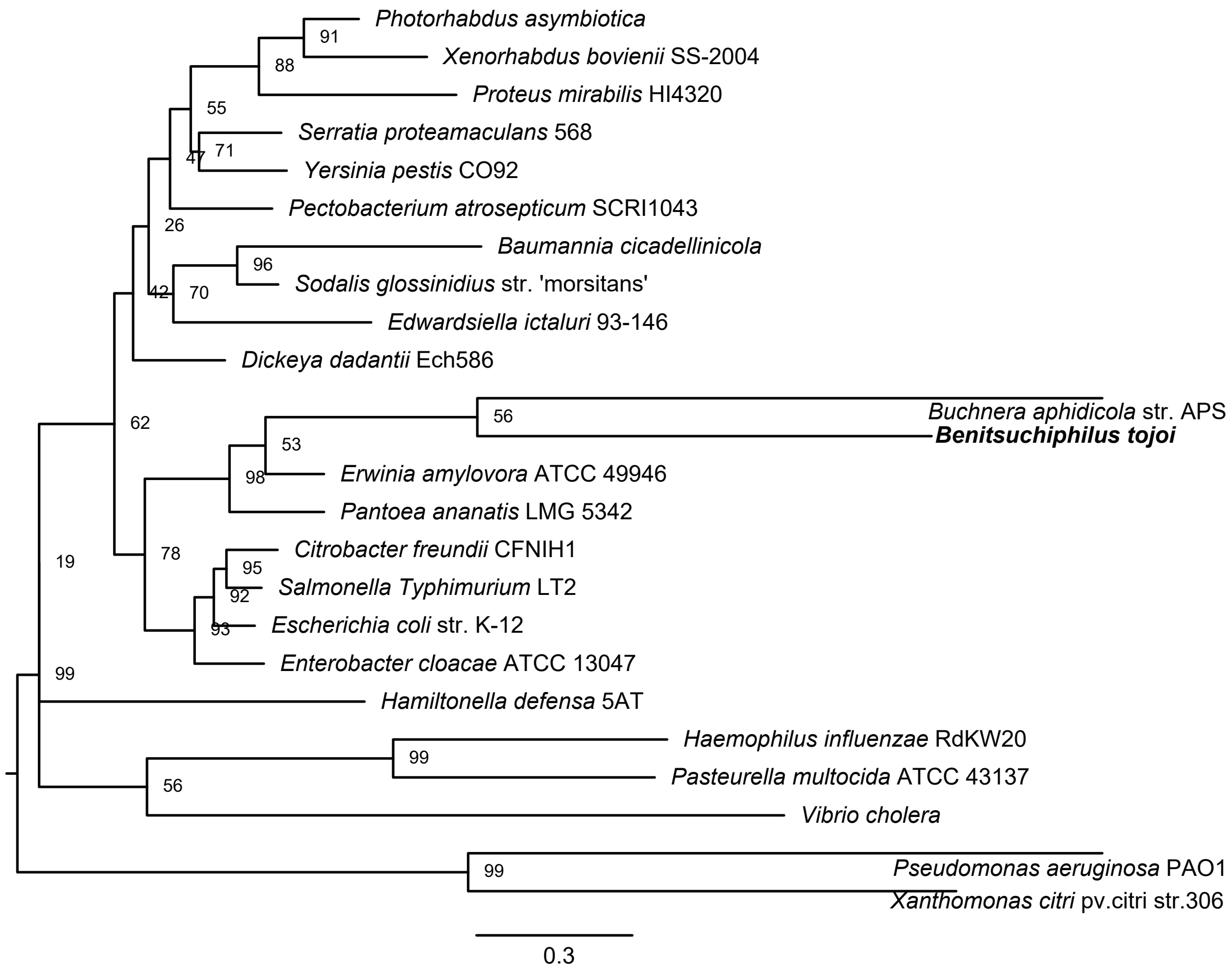

Supplementary Figure S5F. Maximum-likelihood protein phylogenies of ThiL. Numbers on the branches represent the support from 1,000 bootstrap replicates. The scale bar indicates substitutions per site.

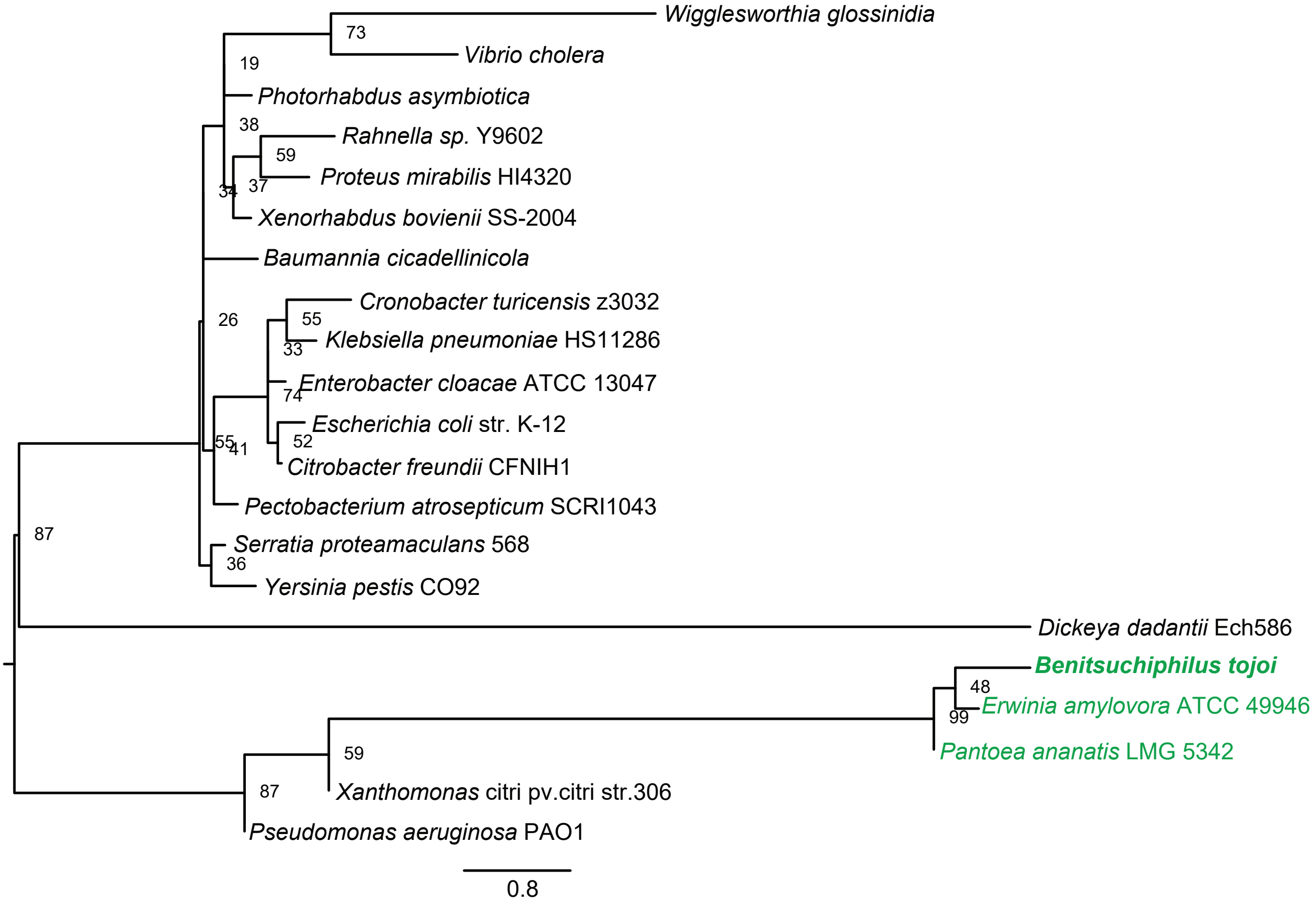

Supplementary Figure S5G. Maximum-likelihood protein phylogenies of ThiS. Green color indicate plasmid origin. Numbers on the branches represent the support from 1,000 bootstrap replicates. The scale bar indicates substitutions per site.

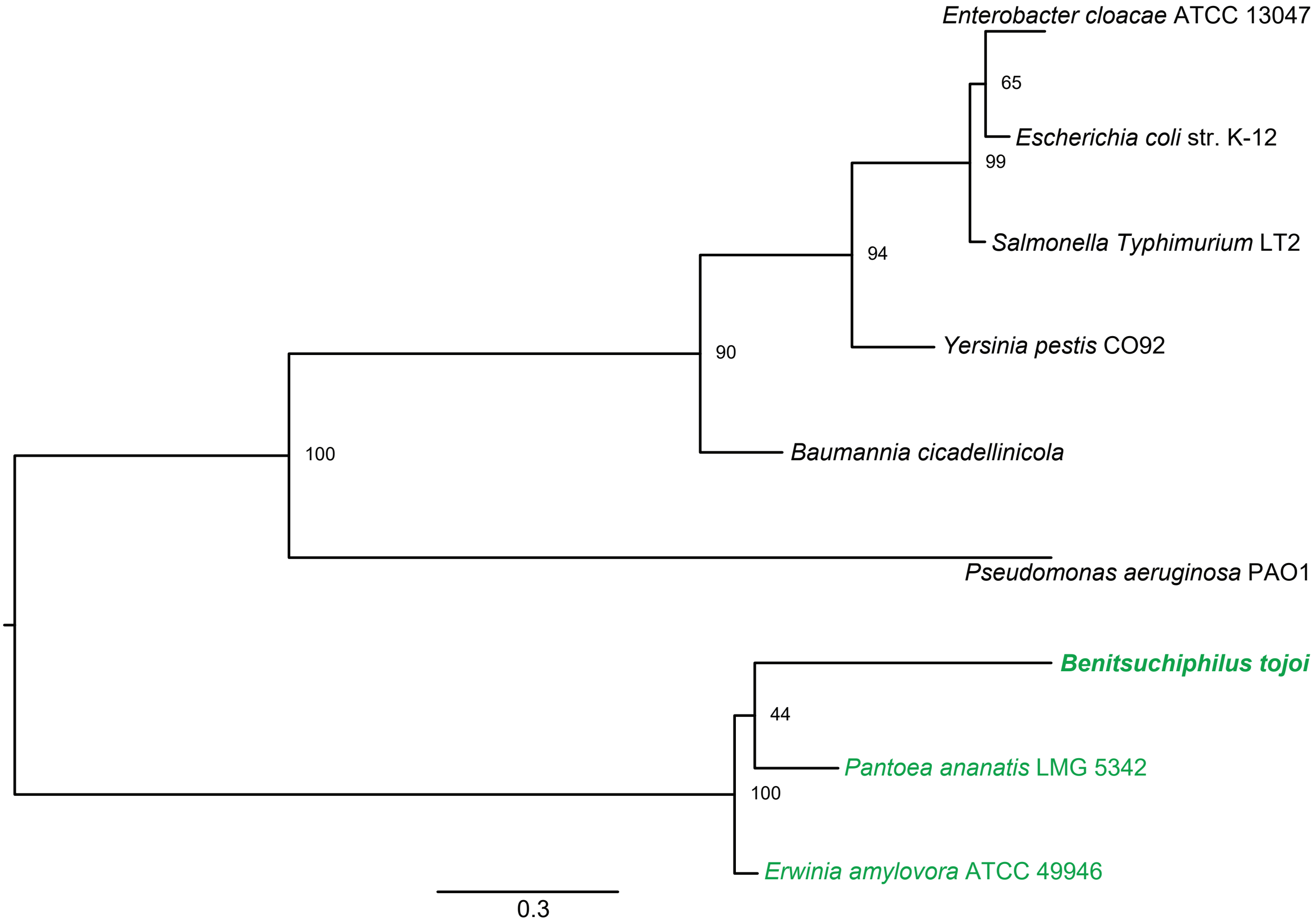

Supplementary Figure S5H. Maximum-likelihood protein phylogenies of ThiG. Green color indicate plasmid origin. Numbers on the branches represent the support from 1,000 bootstrap replicates. The scale bar indicates substitutions per site.
